# Calcitriol stimulates gene expression of cathelicidin antimicrobial peptide in breast cancer cells with different phenotype

**DOI:** 10.1186/s12929-016-0298-4

**Published:** 2016-11-10

**Authors:** Janice García-Quiroz, Rocío García-Becerra, Nancy Santos-Martínez, Euclides Avila, Fernando Larrea, Lorenza Díaz

**Affiliations:** Departamento de Biología de la Reproducción, Instituto Nacional de Ciencias Médicas y Nutrición Salvador Zubirán, Avenida Vasco de Quiroga No. 15, Col. Belisario Domínguez Sección XVI, C.P. 14080 Ciudad de México, México

**Keywords:** Cathelicidin, Breast cancer, LL-37, Calcitriol, Vitamin D

## Abstract

**Background:**

In normal and neoplastic cells, growth-promoting, proangiogenic, cytotoxic and pro-apoptotic effects have all been attributed to cathelicidin antimicrobial peptide (*CAMP*). Nevertheless, little is known about the factors regulating this peptide expression in breast cancer. Herein we asked if the well-known antineoplastic hormone calcitriol could differentially modulate *CAMP* gene expression in human breast cancer cells depending on the cell phenotype in terms of efficacy and potency.

**Methods:**

The established breast cancer cell lines MCF7, BT-474, HCC1806, HCC1937, SUM-229PE and a primary cell culture generated from invasive ductal breast carcinoma were used in this study. Calcitriol regulation of cathelicidin gene expression in vitro and in human breast cancer xenografts was studied by real time PCR. Tumorigenicity was evaluated for each cell line in athymic mice.

**Results:**

Estrogen receptor (ER)α + breast cancer cells showed the highest basal *CAMP* gene expression. When incubated with calcitriol, *CAMP* gene expression was stimulated in a dose-dependent and cell phenotype-independent manner. Efficacy of calcitriol was lower in ERα + cells when compared to ERα- cells (<10 vs. >70 folds over control, respectively). Conversely, calcitriol lowest potency upon *CAMP* gene expression was observed in the ERα-/EGFR+ SUM-229PE cell line (EC_50_ = 70.8 nM), while the highest was in the basal-type/triple-negative cells HCC1806 (EC_50_ = 2.13 nM) followed by ERα + cells MCF7 and BT-474 (EC_50_ = 4.42 nM and 14.6 nM, respectively). In vivo, lower basal *CAMP* gene expression was related to increased tumorigenicity and lack of ERα expression. Xenografted triple-negative breast tumors of calcitriol-treated mice showed increased *CAMP* gene expression compared to vehicle-treated animals.

**Conclusions:**

Independently of the cell phenotype, calcitriol provoked a concentration-dependent stimulation on *CAMP* gene expression, showing greater potency in the triple negative HCC1806 cell line. Efficacy of calcitriol was lower in ERα + cells when compared to ERα- cells in terms of stimulating *CAMP* gene expression. Lower basal *CAMP* and lack of ERα gene expression was related to increased tumorigenicity. Our results suggest that calcitriol anti-cancer therapy is more likely to induce higher levels of *CAMP* in ERα- breast cancer cells, when compared to ERα + breast cancer cells.

## Background

Paradoxical effects have been described for cathelicidin (*CAMP*) in cancer biology. Some studies have shown cytotoxic, antiproliferative and pro-apoptotic effects [1–4], whereas others reported growth-promoting, proangiogenic, prometastatic and invasive-inductive effects of *CAMP* in different malignant-type cells [5–8]. These effects are the result of tissue-specific signaling pathways triggered by *CAMP* in an intra- or extra-cellular manner [9] involving several growth factor receptors [10–12] and/or toll-like receptors [13]. In fact, *CAMP* overexpression has been shown to suppress tumorigenesis in colon and gastric cancer but also to promote development and progression of ovarian, lung and breast cancer [9]. Of note, *CAMP* signalization may activate signaling cascades potentially involved in carcinogenesis, such as those involving mitogen activated kinases, protein kinase C or nuclear factor kappa B. Therefore, overexpression of *CAMP* is generally associated with tumor promotion activity, in a concentration and/or tissue specific fashion. Particularly in the breast, *CAMP* is abundantly produced in both normal and malignant conditions, while its maximum expression has been found among high-grade breast tumors [5]. Interestingly, *CAMP* expression is closely correlated with that of epidermal growth factor receptor 2 (HER2) and with the presence of lymph node metastases in estrogen receptor (ER) + breast tumors, suggesting a prometastatic role for *CAMP* in breast malignancy [14]. The regulatory factors acting upon *CAMP* are not yet completely understood. Normally, the expression of *CAMP* is induced in response to injury or bacterial challenge, resulting in its accumulation in the site of distress. Indeed, inflammatory mediators may induce *CAMP* in some settings [15]; however, this is not always the case, as seen in tumor necrosis factor-α treated trophoblasts and other cell types, where *CAMP* was either not regulated or downregulated by inflammatory cytokines [16, 17]. In humans, there is evidence that the most robust *CAMP* inducer is calcitriol, the vitamin D more active metabolite. This hormone, acting through its nuclear receptor (VDR), transcriptionally induces robust expression of *CAMP* by acting through a vitamin D response element located in its promoter [18]. Calcitriol is well known for its anticancer properties, which are being studied in preclinical and clinical settings. Given the potential pharmacological use of calcitriol for therapeutic purposes in breast cancer patients, herein we thought of importance to investigate the regulatory actions of this hormone upon *CAMP* gene expression under in vitro and in vivo conditions using different phenotypes of breast cancer cells.

## Methods

### Breast cancer cell cultures

The established human breast cancer cell lines MCF7, BT-474, HCC1806, HCC1937 (ATCC, Manassas, VA), SUM-229PE (Asterand, San Francisco, CA), and a primary cell culture generated from invasive ductal breast carcinoma (IDC) [19], were maintained under standard cell culture conditions. For experiments, cells were incubated in the presence of different calcitriol concentrations (0.1–1000 nM, Sigma-Aldrich, St Louis, MO) or its vehicle (0.1 % ethanol) during 24 h. Afterwards, cells were used for RNA isolation. Characterization of the cells was performed by immunocytochemistry in order to analyze the expression of particular molecular markers.

### Immunocytochemistry

Cultured cells were grown on glass coverslips and fixated in 96 % ethanol. Antigen retrieval was done by autoclaving in Retriever EDTA (Bio SB, Santa Bárbara CA, USA). Slides were blocked with immunodetector peroxidase blocker (Bio SB). The following primary antibodies were incubated for 1 h: Anti- ERα (1:250, Bio SB), anti-VDR (1:100, Santa Cruz Biotechnology Inc, CA, USA), anti-HER2 (1:100, Cell Signaling Technology, Beverly, MA) and anti-epidermal growth factor receptor (EGFR, 1:100, Bio SB). After washing, the slides were sequentially incubated with immuno-Detector Biotin-Link and immuno-Detector HRP label (Bio SB) during 10 min each. Staining was completed with diaminobenzidine (DAB) and slides were counterstained with hematoxylin.

### PCR amplifications

Calcitriol effects upon *CAMP* gene expression were studied by extracting total RNA from treated cells and resected tumors using Trizol reagent (Life Technologies, CA, USA). The concentration of RNA was estimated spectrophotometrically at 260/280 nm and a constant amount of RNA (2 μg) was reverse transcribed using a commercial assay (Roche Applied Science, IN, USA). Gene expression of the housekeeping gene β-actin (*ACTB*) was used as internal control. Primers sequences were as follows: *CAMP* [GenBank:NM_004345.3]: forward: tcg gat gct aac ctc tac cg, reverse: gtc tgg gtc ccc atc cat and *ACTB* [GenBank:NM_001101.3]: forward: cca aac cgc gag aag atg a, reverse: cca gag gcg tac agg gat ag. Corresponding probe numbers from the universal probe library (Roche) were: 85 and 64 for *CAMP* and *ACTB*, respectively. Real time PCR amplifications were carried on a LightCycler® 480 Instrument (Roche), according to the following protocol: activation of Taq DNA polymerase and DNA denaturation at 95 °C for 10 min, proceeded by 45 amplification cycles of 10 s at 95 °C, 30 s at 60 °C, and 1 s at 72 °C.

The calcitriol concentration producing 50 % *CAMP* gene expression stimulation (EC_50_) was calculated by non-linear regression analysis using sigmoidal fitting with a sigmoidal dose–response curve by means of the scientific graphing software Origin (OriginLab Corporation, Northampton, MA, USA).

### Induction of tumors in athymic mice

Athymic female BALB/c homozygous, inbred Crl:NU(NCr)-Foxn1nu nude mice (~6 weeks of age) were kept in ventilated cages with bedding of aspen wood-shavings, controlled temperature, humidity and 12:12 light:dark periods. Sterile water and feed (standard PMI 5053 feed) were given *ad libitum*. Endpoints compatible with the scientific objectives of this work were cautiously observed preserving strict animal welfare standards. To evaluate the physical status of each mouse, a scoring method was used which included the following categories: 1) dehydration/loss of appetite, 2) body weight, 3) natural behavior, 4) provoked behavior and 5) inflammation/ulceration in injection site. A value of 1–2 was assigned to the first category while 1–3 was used for the last 4 categories. A total score of 14 indicated wellbeing, while lower scores indicated progressive health deterioration. A score < 9 was an automatic endpoint. Tumorigenicity was evaluated for each cell line used in this study by subcutaneous injection of 2.0 × 10^6^ cells in 0.1 mL of sterile saline solution into the upper part of the posterior limb of each mouse.

### Therapeutic protocol

When the tumors reached a palpable mass (~ 3 mm), mice were separated in two groups: control and calcitriol-treated (calcitriol Geldex, GELpharma, México, 12.5 μg/kg of body weight i.p. in 100 μL once a week during 3 weeks). IDC and HCC1806 cells were used to xenograft mice (total mice = 22; 11 for each cell line). Body weights and tumor sizes were measured thrice weekly throughout the experiment. Tumor volume was calculated using the standard formula (length x width^2^)/2, where length is the largest dimension and width the smallest dimension perpendicular to the length. Tumors were measured with a caliper always by the same person. Relative tumor volume was calculated for each tumor by dividing the tumor volume on day 21 by that on day 0 (which corresponded to the tumor volume in the first day of treatment, and was set to one). After sacrifice, tumors were excised and processed for RNA extraction.

### Statistical analysis

Statistical differences for in vitro dose-response assays were determined by one-way ANOVA followed by appropriate post-hoc tests using a specialized software package (SigmaStat, Jandel Scientific). For in vivo comparisons between control and calcitriol-treated groups Student’s t-test was used. Differences were considered statistically significant at *P* < 0.05.

## Results

### Characterization of the cells used in this study

Expression of VDR, ERα, HER2 and EGFR for each cell line is depicted in Table 1.

**Table 1:** Cell characterization by immunocytochemistry

	MCF7	BT-474	SUM-229PE	HCC1806	HCC1937	IDC
VDR	+	+	+	+	+	+
ERα	+	+	–	–	–	–
HER2	–	+	S	–	–	–
EGFR	–	+	+	+	+	+

Expression of vitamin D receptor (VDR), estrogen receptor-α (ERα), epidermal growth factor receptor (EGFR) and epidermal growth factor receptor 2 (HER2) are depicted. S = Only slight expression was detected

Also, the functionality of the VDR was corroborated by the calcitriol-dependent induction of CYP24A1 gene expression (Table 2).

**Table 2:** Stimulation of CYP24A1 gene expression by calcitriol

Cell line	Mean ± SD (folds over control)
HCC1937	5.69 ± 0.94
IDC	47.1 ± 18.9
BT-474	83.23 ± 18.01
MCF7	220.42 ± 60.81
HCC1806	7579 ± 1711
SUM-229PE	31181 ± 6192

Depicted cell lines were incubated with 10 nM calcitriol during 24 h and afterwards RNA was extracted and qPCR performed. Control was normalized to one, results are expressed as fold induction over control

### Calcitriol induces *CAMP* gene expression in cultured breast cancer cells of different phenotype, but more strongly in ERα- cells

**Fig. 1:**
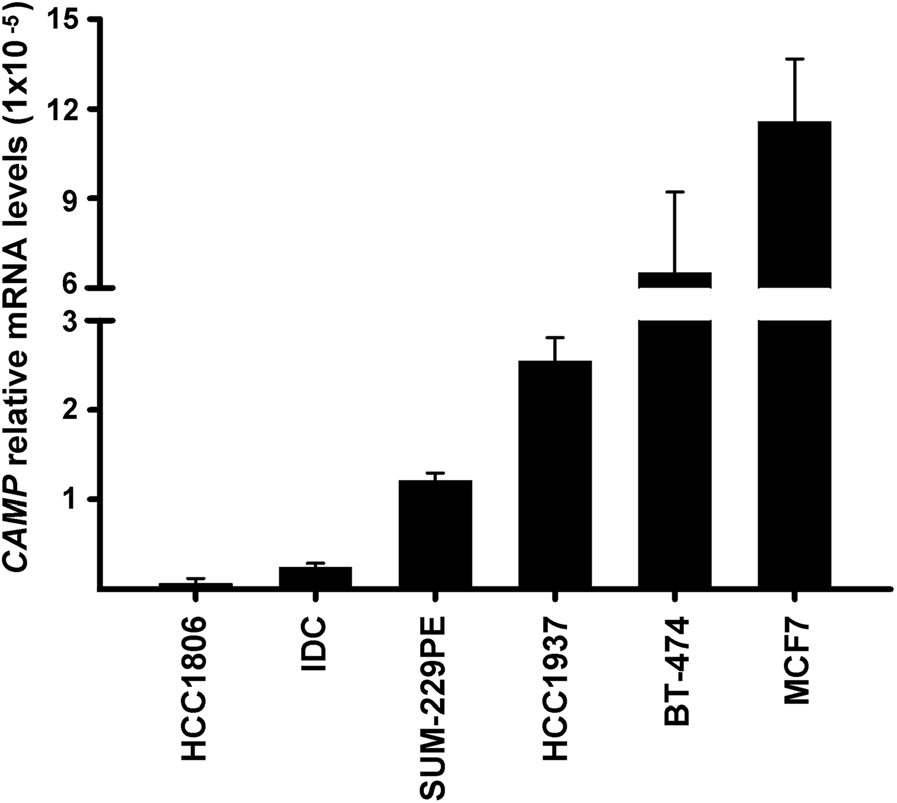
Basal *CAMP* gene expression. Basal *CAMP* gene expression was evaluated in several breast cancer cell lines with different phenotype. Data are depicted as the mean ± SD. *N* = 3. Results were normalized against *ACTB* mRNA expression

**Fig. 2:**
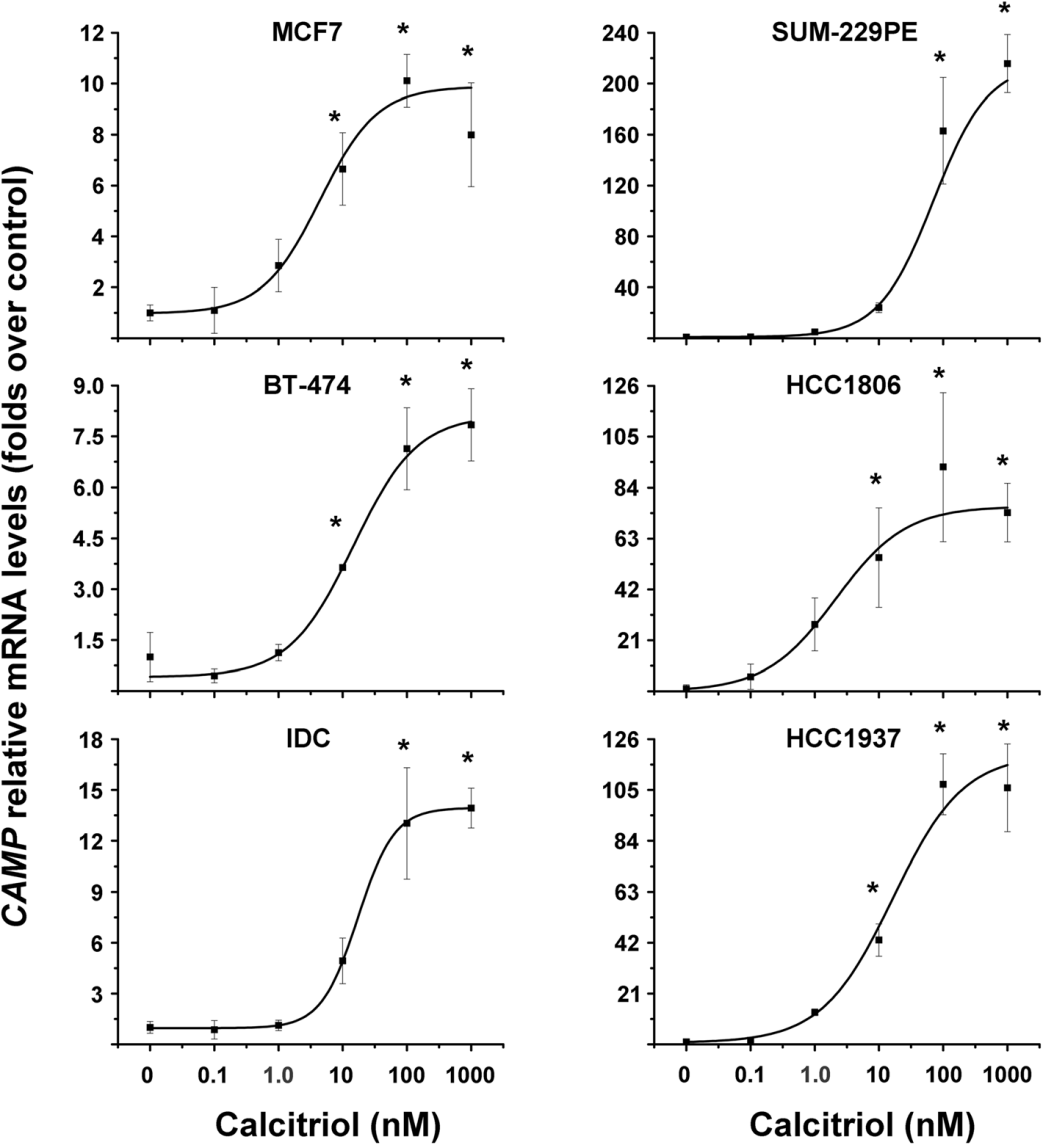
*CAMP* is transcriptionally upregulated by calcitriol in different human breast cancer cell lines. Cells were incubated in the presence of different calcitriol concentrations during 24 h. Afterwards cells were processed for qPCR. *CAMP* mRNA levels were obtained by normalizing against *ACTB* mRNA expression. Vehicle values were set to one. *N* = 3, *P < 0.05 vs. control

Differential basal *CAMP* gene expression was observed depending on the cell line. In particular, ERα + breast cancer cells showed the highest basal *CAMP* gene expression, while the lowest was obtained in ERα- cells (Fig. 1). On the other hand, in all cell lines tested, a calcitriol dose-dependent stimulation of *CAMP* gene expression was observed (Fig. 2). In particular, in the ERα-/EGFR+ cell line SUM-229PE, calcitriol, at the highest concentration tested, showed the greatest efficacy in terms of stimulating *CAMP* gene expression (>200 folds over control). Meanwhile, in the basal-type/triple-negative cell lines HCC1937 and HCC1806 calcitriol increased *CAMP* gene expression by approximately 70–100 folds over the control. In contrast, ERα + cells MCF7 and BT-474 responded more moderately to calcitriol (<10 folds over control, Fig. 2). Based on the EC_50_ values, the potency/sensibility of calcitriol upon *CAMP* gene expression was: HCC1806 > MCF7 > BT-474 > HCC1937 > IDC > SUM-229PE (Table 3).

**Table 3:** Stimulatory concentrations (EC)_50_ values for calcitriol upon *CAMP* gene expression in breast cancer cells

Breast cancer cell line	EC_50_ (nM)
HCC1806	2.13
MCF7	4.42
BT-474	14.6
HCC1937	16.3
IDC	17.1
SUM-229PE	70.8

The effect of calcitriol upon stimulation of cathelicidin gene expression was evaluated in different types of breast cancer cells

### In a xenograft model of breast cancer, calcitriol induced *CAMP* gene expression

**Fig. 3:**
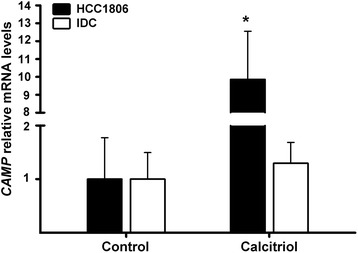
*CAMP* gene expression is stimulated by calcitriol in xenografted tumors. IDC (*white bars*) and HCC1806 (*black bars*) cells were inoculated in athymic mice. After tumor onset calcitriol was administered once per week during 3 weeks. Mice were sacrificed and tumors were collected to evaluate *CAMP* gene expression by qPCR. Results are depicted as the mean ± SEM. Controls were set to one *N* ≥ 5; *P < 0.05 vs. control

**Fig. 4:**
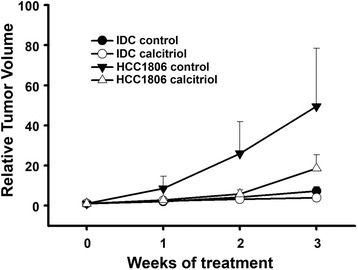
Calcitriol reduces tumor growth in mice xenografted with human breast cancer cells. HCC1806 cells (*triangles*) and IDC cells (*circles*) were subcutaneously injected in athymic mice, which were treated without (*black*) or with 12.5 μg/kg calcitriol (*white*) during three weeks. Relative tumor volume is shown as the mean ± SEM. *N* ≥ 5

We first tested tumorigenicity of all cancer cell lines in a murine model. Under the conditions of this study, only HCC1806 and IDC readily formed tumors. We observed that lower basal *CAMP* gene expression and lack of ERα positivity were cell features related to increased tumorigenicity. Therefore, *CAMP* gene expression in vivo studies were carried out in HCC1806 and IDC tumors. Considering the in vitro calculated potency of calcitriol upon *CAMP* gene expression, which was higher in HCC1806 compared to IDC (2.13 nM vs. 17.1 nM, respectively), and the greatest efficacy of calcitriol to induce its canonic transcriptional target CYP24A1 in HCC1806 vs. IDC cells (7579 vs. 47 folds over the control, respectively), the results observed in vivo mirrored in vitro findings. Indeed, calcitriol treatment of mice xenografted with HCC1806 cells significantly stimulated tumoral *CAMP* gene expression compared to tumors from untreated mice. In contrast, in IDC-grafted mice calcitriol did not significantly affect tumoral *CAMP* gene expression (Fig. 3). In both xenotransplanted mouse models calcitriol reduced, although not significantly, the relative tumor volume (Fig. 4).

### Serum levels of total calcium and body weight in calcitriol-treated xenotransplanted mice

Serum samples from each experimental group were pooled. As expected, serum total calcium was higher in calcitriol treated mice compared to controls (10.6 vs. 9.9 mg/dL); however, no signs of hypercalcemia were detected (e.g. dehydration, weight loss). Final body weights were not significantly different among the treated and control groups.

## Discussion

Cathelicidin is produced by the human mammary gland and is found in human milk exerting antimicrobial activity [20], which highlights the important physiological role of this antimicrobial peptide in the newborn innate immune defense during lactation. Nevertheless, in a pathological scenario of the breast, cathelicidin effects are controversial since it has been implicated in tumor-suppressive activities, but also in promoting tumor growth and vascularization [5, 8, 10, 21]. Given that calcitriol, a recognized antineoplastic hormone, is the most known robust inducer of *CAMP* expression in humans, herein we studied the regulatory actions of this compound upon *CAMP* expression in vitro and in vivo in different types of breast cancer cells. Our in vitro results showed that calcitriol, at clinically achievable concentrations, was able to significantly stimulate *CAMP* gene expression in a cell-type specific manner. Indeed, pharmacological phase I clinical studies involving subjects affected with cancer have demonstrated that therapeutic calcitriol may reach peak blood levels of 3–16 nM [22, 23] and herein, with the exception of the cell line SUM-229PE, the EC_50_ of calcitriol upon *CAMP* gene expression values ranged between 2.13 and 17.1 nM. The fact that in SUM-229PE cells the EC_50_ value was very high might be related to the observation that in this cell line calcitriol showed the highest potency to stimulate CYP24A1, the enzyme that inactivates calcitriol, which most probably resulted in lesser bioavailability of calcitriol in these cells. Whereas calcitriol potency was apparently not related to the cell phenotype, this secosteroid clearly showed a greater efficacy to increase *CAMP* gene expression in ERα- cells. In fact, in HCC1937, HCC1806 and SUM-229PE cells calcitriol increased *CAMP* gene expression from 70 folds to more than 200 folds over control, in clear contrast with ERα + cells where this stimulus was less than 10 folds over control. These results suggest that ERα- breast tumors are more likely to produce greater amounts of *CAMP* in response to therapeutic calcitriol, compared to ERα + tumors. Nevertheless, it should be noted that cells with higher basal *CAMP* gene expression were in fact ERα+, which might probably indicate that in this cell phenotype a steady state in *CAMP* gene expression has already been reached. On the other hand, in the in vivo model used herein ERα + cells did not readily formed tumors, probably due to the lack of estrogen supplementations to mice. Of the tumorigenic cell lines, triple negative tumors from the highly undifferentiated HCC1806 cells expressed significantly more *CAMP* in response to calcitriol compared to IDC tumors, in accordance to the potency of calcitriol upon *CAMP* gene expression observed in vitro. Probably, the time and dose used herein to treat mice with calcitriol might account on the lack of statistical significance found upon tumor volume in this study.

Regarding *CAMP* biological actions in tumoral cells, it is noteworthy to mention that *CAMP* expression has been closely correlated to HER2 [14] and has been shown to transactivate EGFR [24, 25], which may explain why cancer cells exposed to the *CAMP* active peptide LL-37 show increased cell proliferation and invasion [8]. Similarly, in animal models *CAMP* treatment promoted tumor growth and metastasis [14]. Nevertheless, the fact that the highest *CAMP* levels have been found in breast tumors of greater malignancy grade [5], together with the observation of increased *CAMP* expression in blood of breast cancer patients compared to healthy women [26], strongly encourages to explore the biological actions of *CAMP* in breast tumor progression. In this regard, binding of LL-37 to type I insulin-like growth factor receptor in different types of breast cancer cells has resulted in intra-cellular signaling activation and increased migratory and invasive potential of malignant cells [10]. While additional studies of *CAMP* effects on breast cancer biology await to be undertaken, the calcitriol-mediated induction of *CAMP* gene in cancerous tissues has been shown in B-cell lymphomas, acute myeloid leukemia, colon, prostate, endometrial and ovarian cancer cell lines [27–31]. Of particular interest is the observation that in early premalignant and fully malignant breast cells a similar stimulatory effect of a calcitriol analogue upon *CAMP* gene expression has been observed [32]. However, to our knowledge this is the first study to show a differential *CAMP* gene expression profile after calcitriol stimulation depending of the cell type phenotype. Given that calcitriol is an antineoplastic drug under intense investigation for therapeutic purposes, the results in this study may help to translate calcitriol therapy into the clinic. Since *CAMP* may regulate tumorigenesis and/or cell proliferation, more studies are needed in order to clarify exogenous calcitriol-dependent *CAMP* synthesis and biological actions in breast tumors with different phenotype.

## Conclusions

Breast cancer cells showed differential basal *CAMP* gene expression depending on the cell phenotype: ERα + breast cancer cells showed the highest while ERα- the lowest. Independently of the cell phenotype, calcitriol provoked a concentration-dependent stimulation on *CAMP* gene expression, showing greater potency in the triple negative HCC1806 cell line. Efficacy of calcitriol was lower in ERα + cells when compared to ERα- cells in terms of stimulating *CAMP* gene expression. Lower basal *CAMP* and lack of ERα gene expression were related to increased tumorigenicity. Our results suggest that calcitriol anti-cancer therapy is more likely to induce higher levels of *CAMP* in ERα- breast cancer cells when compared to ERα + breast cancer cells.

## Abbreviations

CAMP: Cathelicidin
HER2: Epidermal growth factor receptor 2
VDR: Vitamin D receptor
